# Age-Related Changes in Topological Properties of Individual Brain Metabolic Networks in Rats

**DOI:** 10.3389/fnagi.2022.895934

**Published:** 2022-05-13

**Authors:** Xin Xue, Jia-Jia Wu, Bei-Bei Huo, Xiang-Xin Xing, Jie Ma, Yu-Lin Li, Dong Wei, Yu-Jie Duan, Chun-Lei Shan, Mou-Xiong Zheng, Xu-Yun Hua, Jian-Guang Xu

**Affiliations:** ^1^School of Rehabilitation Science, Shanghai University of Traditional Chinese Medicine, Shanghai, China; ^2^Department of Rehabilitation Medicine, Yueyang Hospital of Integrated Traditional Chinese and Western Medicine, Shanghai University of Traditional Chinese Medicine, Shanghai, China; ^3^Engineering Research Center of Traditional Chinese Medicine Intelligent Rehabilitation, Ministry of Education, Shanghai, China; ^4^Department of Traumatology and Orthopedics, Yueyang Hospital of Integrated Traditional Chinese and Western Medicine, Shanghai University of Traditional Chinese Medicine, Shanghai, China

**Keywords:** aging, PET, graph theory, metabolic networks, rich-club organization

## Abstract

Normal aging causes profound changes of structural degeneration and glucose hypometabolism in the human brain, even in the absence of disease. In recent years, with the extensive exploration of the topological characteristics of the human brain, related studies in rats have begun to investigate. However, age-related alterations of topological properties in individual brain metabolic network of rats remain unknown. In this study, a total of 48 healthy female Sprague–Dawley (SD) rats were used, including 24 young rats and 24 aged rats. We used Jensen-Shannon Divergence Similarity Estimation (JSSE) method for constructing individual metabolic networks to explore age-related topological properties and rich-club organization changes. Compared with the young rats, the aged rats showed significantly decreased clustering coefficient (*Cp*) and local efficiency (*E*_*loc*_) across the whole-brain metabolic network. In terms of changes in local network measures, degree (*D*) and nodal efficiency (*E*_*nod*_) of left posterior dorsal hippocampus, and *E*_*nod*_ of left olfactory tubercle were higher in the aged rats than in the young rats. About the rich-club analysis, the existence of rich-club organization in individual brain metabolic networks of rats was demonstrated. In addition, our findings further confirmed that rich-club connections were susceptible to aging. Relative to the young rats, the overall strength of rich-club connections was significantly reduced in the aged rats, while the overall strength of feeder and local connections was significantly increased. These findings demonstrated the age-related reorganization principle of the brain structure and improved our understanding of brain alternations during aging.

## Introduction

The brain is a dynamic system that can be modeled as a complex network of structurally interconnected elements (Bullmore and Sporns, 2009). Most studies have shown that brain function depends on the topologic organization of the entire brain network, rather than individual regions or connections (Sporns et al., 2005; Moon et al., 2015, 2017). There are normal physiologic and psychologic changes that occur as people age, such as gait and mobility problems (Cruz-Jimenez, 2017) and decline in executive functions and memory (Kirova et al., 2015). Characterizing changes of the brain network is invaluable to increasing our understanding of age-related decline, even in the absence of disease.

Using graph theoretical tools, the brain can be modeled as a series of interactive networks composed of nodes and edges. Brain regions are nodes of the network, and the structural or functional connections between nodes are edges of the network (Kaplan et al., 2019). Global structure parameters can reveal the organization of the entire network, while regional structure parameters can capture the contributions of brain regions (Sporns et al., 2007). It is worth noting that there is a robust hub structure in the brain network, which plays a central role in the whole network to promote information integration and global communication (van den Heuvel and Sporns, 2011, 2013). They are considered as “brain hubs” and together form a higher-level of organization called “rich-club” (Colizza et al., 2006; Zamora-López et al., 2010). More importantly, the brain regions in the rich-club structure are more likely to be interconnected.

In recent years, with the extensive exploration of the topological characteristics of human brain, rodent studies have begun to shed light on the function of such complex network organization. Some studies have confirmed that there is an obvious rich-club structure in the functional brain networks of rats, just as in the human brain (Liang et al., 2018). However, studies on topological properties and rich-club architecture of metabolic brain networks in rodents are still very scarce and need to be further investigated.

(18F) Fluorodeoxyglucose with positron emission tomography (18F-FDG PET) is a valuable tool for detecting brain structural changes. Currently, most studies on constructing metabolic networks are based on group-level data. In the current study, we collected 18F-FDG PET data in the young and aged rats and constructed individual metabolic networks. Using systematically graph theory methods to assess age-related topological properties and rich-club organization changes occurring in the rat individual brain metabolic networks. Exploring the age-related reorganization mechanism of the brain network is of great clinical significance for understanding and identifying the functional decline and disease progression caused by aging.

## Materials and Methods

### Animals

In this study, a total of 48 healthy female Sprague–Dawley (SD) rats were used, including 24 young rats and 24 aged rats. The young rats were 8 weeks old and weighed 180–200 g, and the old rats were 18 months old and weighed 350–380 g. All rats were provided adequate water and food, and fed in temperature-controlled laboratory with a 12-h light/12-h dark cycle for 1 week. All rats were obtained from the Shanghai Slack Laboratory Animal Limited Liability Company (Shanghai, China). Prior to the formal study, this protocol was approved by the Animal Ethical Committee of Shanghai University of Traditional Chinese Medicine.

### 18F-Fluorodeoxyglucose With Positron Emission Tomography/CT Acquisition

Positron emission tomography imaging was carried out on a dedicated small animal PET/CTR4 bed (Siemens Inc., United States). Following an overnight fast, each rat was injected through the tail vein with 0.5 mCi 18F-FDG 40 min before scanning, and anesthetized with 5% halothane gas inhalation at induction, followed by a 1.5% halothane gas maintenance dose during scanning. The attenuation correction for the 18F-FDG datasets was automatically performed to obtain a 128 × 128 matrix at the end of the acquisition period. The parameters of PET/CT acquisition were as follows: current = 500 μA, spherical tube voltage = 80 kV, and time = 492 s.

### Data Preprocessing

The ImageJ software (Image Processing and Analysis in Java, National Institutes of Health, Bethesda, MD, United States) was used for data format conversion. The Statistical Parametric Mapping 8 toolbox (SPM 8^1^) was used for data preprocessing (Ceccarini et al., 2013). First, converting PET/CT images from the DICOM-format to the NIFTI-format. Second, we hand-painted masks to obtain the skull-stripped brain images. Third, according to a standard rat brain template (Schwarz et al., 2006), the orientation of these images was modified by adjusting parameters, and the origin correction was completed. Fourth, the image voxels were magnified 10 times to meet the algorithm requirements in SPM8 (Choi et al., 2015). Fifth, each brain PET image was normalized to the standard brain space (Schwarz et al., 2006). Finally, the 18F-FDG value of each voxel was divided by the mean value of the entire brain to obtain the globally normalized PET image maps (Hou et al., 2020; Wang et al., 2020).

### Individual Metabolic Network Construction

Jensen-Shannon Divergence Similarity Estimation (JSSE) method was used for constructing individual metabolic networks (Li et al., 2021, 2022). Briefly, this approach defined the similarity between the probability distribution of the standard uptake values of a group of voxels in a region of interest (ROI) with that in another ROI as the metabolic connectivity between any pair of brain regions in an individual brain metabolic network (Li et al., 2022). In the present study, we defined the nodes of the brain network by parcellating the brain into different ROIs according to the standard rat brain template (Schwarz et al., 2006). Then, the voxel intensity of each brain region was extracted from the globally normalized FDG uptake maps, and the probability density function of the corresponding region was estimated by kernel density estimation (Duong, 2007). Next, we calculated the Kullback–Leibler divergence (KLD) (relative entropy) of each pair of brain regions, according to the mathematical equation (Wang et al., 2020):


$$D_{KL}\left(P||Q\right)=\int_{x}\left(P\left(x\right)\log\frac{P\left(x\right)}{Q\left(x\right)}+Q\left(x\right)\log\frac{Q\left(x\right)}{P\left(x\right)}\right)dx$$

where *P* and *Q* represent the probability density functions of voxel intensities in two brain regions, the “| |” operator indicates “divergence.” The higher similarity of the probability density functions between two ROIs, the nearer consistent functional activity levels in two brain regions (Lin et al., 2021). However, the KLD is not symmetric. Thus, referring to relevant literature (Li et al., 2021), we defined the edges of the brain network as the metabolic connections by JSSE, according to the mathematical equation:


$$D_{JS}\left(P||Q\right)=\frac{1}{2}\left[D_{KL}\left(P||M\right)+D_{KL}\left(Q||M\right)\right]$$

where *M* = 0.5 × (*P* + *Q*) and *D*_*KL*_( | ) are the KLD. Accordingly, we applied the JS divergence (JSD) to construct the adjacency matrix, where the corresponding element represented the metabolic connection strength. Finally, the 96 × 96 metabolic correlation adjacency matrix was referred as the individual brain metabolic network.

### Network Topology Metrics

The GRETNA toolbox^2^ was used to perform the graph theory analysis. Each absolute matrix was restricted at a range of densities (0.1–0.4, interval of 0.01) to generate a set of binary undirected networks. And the topological metrics of metabolic brain networks were calculated at each density.

To describe the global topologic properties of the individual metabolic network, the following parameters were calculated: path length (*Lp*), clustering coefficient (*Cp*), global efficiency (*E*_*glob*_), local efficiency (*E*_*loc*_), and small-worldness. In a network, the *Lp* is the average shortest *Lp* overall pair of nodes in this network. First, converting the metabolic connection matrix was transformed to the connection length matrix. The Dijkstra algorithm was then used to calculate the shortest distance between pairs combinations of brain regions from the connection length matrix (Watts and Strogatz, 1998). The *Cp* of each node can be defined as the ratio of the number of edges that exist between any two neighbors of the node to the number of all possible edges between such neighbors. The *Cp* value of the network can be obtained by averaging the *Cp* values of all nodes in the network (Watts and Strogatz, 1998). The *E*_*glob*_ is defined as the estimation of the efficacy of information transfer between two nodes that are far apart in the network (Latora and Marchiori, 2001). The *E*_*loc*_ plays a similar evaluation role to *Cp*, which refers to the average efficiency of local subgraphs (Latora and Marchiori, 2001). Small-worldness index (σ) is defined as the value of normalized *Cp* (γ) divided by normalized *Lp* (λ) (Humphries and Gurney, 2008; Li and Huang, 2020). By comparing the *Cp* and *Lp* to the mean *Cp*_*rand*_ and *Lp*_*rand*_ of 5,000 random networks, the γ and λ were calculated.

For the regional topologic prosperities, the following parameters were calculated at each node of each graph: betweenness centrality (*BC*), degree (*D*), and nodal efficiency (*E*_*nod*_). The *BC* is the number of shortest-paths through the network that pass through the node (Stam and Reijneveld, 2007). A node’s *D* is defined as the number of edges connected to the node, reflecting the importance of the node in the network (Rubinov and Sporns, 2010). The *E*_*nod*_ represents the efficiency of information transfer between the neighbors of a particular node (Qba et al., 2021).

### Rich-Club Organization

Rich-club organization for each rat at a range of densities (0.1–0.4, interval of 0.01) was estimated using the GRETNA toolbox. The rich-club phenomenon is defined as the tendency of high connectivity between high-degree nodes, such as hub regions (Colizza et al., 2006). The calculation formula is:


$$\Phi_{norm}\left(k\right)=\frac{\Phi \left(k\right)}{\Phi_{rand}\left(k\right)}$$

where the rich-club coefficient Φ(*k*) is calculated by the following three steps: First, a rich-club subnetwork was obtained by extracting the nodes with degree greater than predefined *k* and the edges among them. Next the total number of edges (E) and nodes (n) within rich-club subnetwork was counted. Third, the Φ(*k*) is calculated as the ratio of E to number of all possible connections among this set of nodes [*n* × (*n*−1)] (McAuley et al., 2007; van den Heuvel and Sporns, 2011). In this study, we normalized Φ(*k*) relative to 1,000 random networks with the same size and similar connectivity distribution as the real network. The overall random rich-club coefficient [Φ*_*rand*_*(*k*)] was computed as the average Φ(*k*) over the 1,000 random networks. The brain network would be treated as a rich-club organization when the normalized rich-club coefficient [Φ*_*norm*_*(*k*)] is greater than 1.

### Definition of Hub Regions and Three Connection Types

Nodes with high degree (*k*) were classified as network hubs. In this study, considering the aging-related brain alterations in aged rats, hub regions were defined only by nodes with high degree of young rats (Shu et al., 2017). We selected the top 10 (10%) nodes with the highest *k* as hub regions based on the average individual metabolic network of young rats (Wang et al., 2021). After dividing hub brain regions and non-hub brain regions, edges of the metabolic network were classified into three connection types: (1) rich-club connections, which link hubs and hubs; (2) feeder connections, which link hubs and non-hubs; and (3) local connections, which link non-hubs and non-hubs. Then, the sum of the edge weights for each connection type was calculated as the connectivity strength (Yan et al., 2018).

### Statistical Analysis

To further simplify the statistical analysis, the area under the curve (AUC) over the density range of each topological metric was calculated and then employed as a scalar value. Statistical analyses were performed employing two-sample *t*-tests for group comparison. For the global network analysis and the connectivity strength analysis, the 0.05 level (*p* < 0.05) was accepted for statistical significance. And the significance threshold for regional network analysis was set at *p* < 0.05 and adjusted for multiple comparisons using Bonferroni correction.

## Results

### Animals

All rats were in a normal and stable active state before scanning. We finally used the data of 23 aged rats and 24 young rats for statistical analysis, because one of the 24 aged rats was excluded for poor image quality.

### Network Topological Properties

**FIGURE 1:**
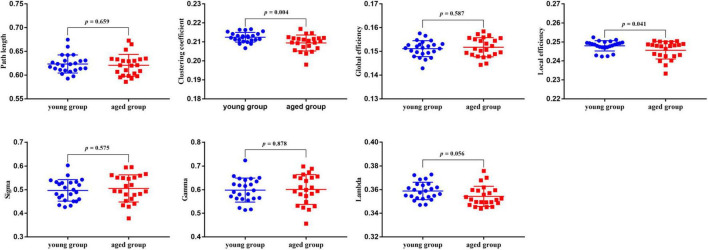
The results of global properties between the young rats and aged rats. And *p* < 0.05 indicates significant differences.

**FIGURE 2:**
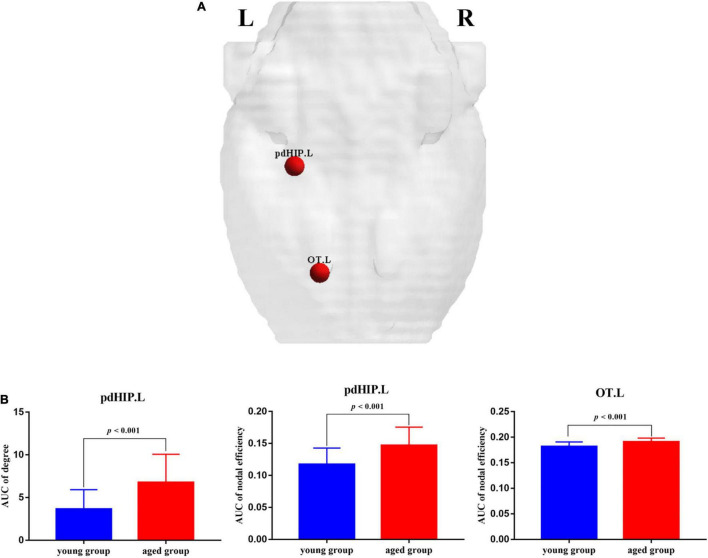
The nodes showed significant changes in regional properties between the young rats and the aged rats. **(A)** Red spheres indicate nodes with increased nodal properties in the aged rats. **(B)** The bar plots display the mean (standard error) of degree and nodal efficiency values of pdHIP.L and OT.L for each group. pdHIP.L, left posterior dorsal hippocampus; OT.L, left olfactory tubercle.

Compared with the young rats, *Cp* and *E*_*loc*_ were significantly lower in the aged rats (*p* < 0.05) (Table 1 and Figure 1). Out of the 96 regions in the aged rats, left posterior dorsal hippocampus showed significantly higher *D* and *E*_*nod*_, and left olfactory tubercle had significantly higher *E*_*nod*_ than those of the young rats. However, no node was found to be significantly different in terms of *BC* (Table 2 and Figure 2).

**TABLE 1:** Intergroup differences of global network properties.

Global network properties	Young group (*n* = 24)	Aged group (*n* = 23)	*p*-values
Path length	0.623 ± 0.019	0.621 ± 0.023	0.659
Clustering coefficient	0.212 ± 0.003	0.209 ± 0.004	0.004
Global efficiency	0.151 ± 0.003	0.152 ± 0.004	0.587
Local efficiency	0.248 ± 0.003	0.246 ± 0.005	0.041
σ	0.497 ± 0.045	0.505 ± 0.057	0.575
γ	0.598 ± 0.051	0.601 ± 0.063	0.878
λ	0.359 ± 0.007	0.354 ± 0.008	0.056

*Data are expressed as the mean ± SD.*

**TABLE 2:** Intergroup differences of regional network properties.

Brain regions	*p*-Values		
	Betweenness centrality	Degree	Nodal efficiency
**Aged rats > Young rats**			
Posterior dorsal hippocampus_L	–	<0.001	<0.001
Olfactory tubercle_L	–	–	<0.001

### Rich-Club Organization

**FIGURE 3:**
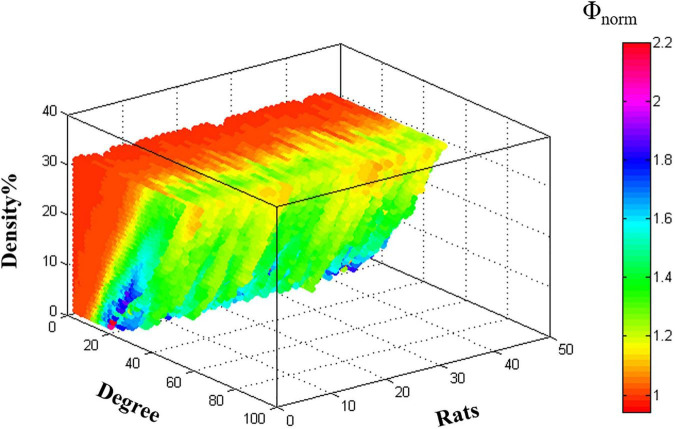
The characteristic rich-club organization of individual metabolic networks in young rats and aged rats.

The rich-club organization of individual metabolic network [Φ*_*norm*_*(*k*) > 1] was found for both young rats and aged rats under the specific range of density (Figure 3).

### Rich-Club Regions

**FIGURE 4:**
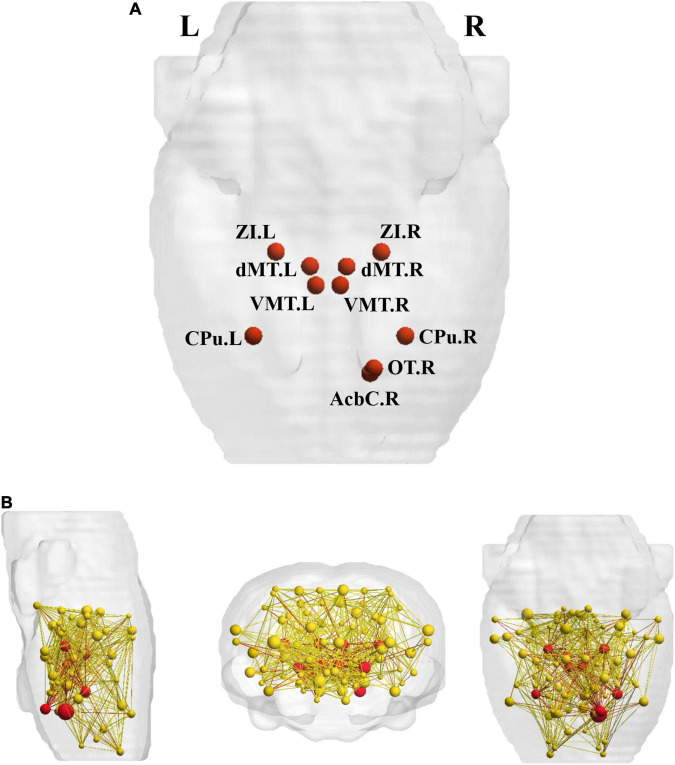
The rich-club regions and three types of connections. **(A)** Ten rich-club members (red nodes) across the young group; **(B)** the red nodes and the yellow nodes represent hub and non-hub regions respectively. The dark red lines represent rich-club connections; the light red lines represent feeder connections and the yellow lines represent local connections. Cpu, caudate putamen; dMT, dorsal midline thalamus; VMT, ventromedial thalamus; ZI, zona incerta; Acbc, nucleus accumbens core; ON, olfactory tubercle.

Ten hub regions were identified, including bilateral caudate putamen (CPu), dorsal midline thalamus (dMT), ventromedial thalamus (VMT), zona incerta (ZI), as well as right nucleus accumbens core (Acbc) and olfactory tubercle (ON) (Figure 4).

### Group Differences in Metabolic Connectivity

**FIGURE 5:**
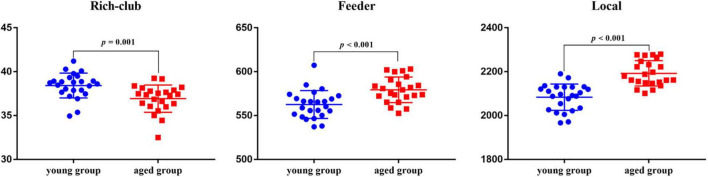
Between-group differences of overall strength in rich-club, feeder, and local connections.

Relative to the young rats, the aged rats exhibited significantly increased overall strength in feeder and local connections (feeder: *p* < 0.001; local: *p* < 0.001), whereas rich-club connections strength decreased significantly in the aged rats (*p* = 0.001) (Figure 5).

## Discussion

Interest in characterizing functional, structural, and metabolic brain alterations during normal aging is growing. Understanding the underlying mechanisms across the whole-brain networks may help us better understand age-related changes. In this study, a graph-theoretical approach was applied to study the topological organization of individual brain metabolic network in the young rats and aged rats. There were three main results: (1) compared with the young rats, *Cp* (*p* = 0.004) and *E*_*loc*_ (*p* = 0.041) significantly decreased in the aged rats; (2) for regional network properties, the aged rats showed significantly higher *D* and *E*_*nod*_ in left posterior dorsal hippocampus (*p* < 0.001), and *E*_*nod*_ in left olfactory tubercle (*p* < 0.001); and (3) relative to the young rats, the overall strength of rich-club connections was significantly reduced (*p* = 0.001) in the aged rats, while the overall strength of feeder and local connections was significantly increased (*p* < 0.001).

Abnormalities in functional segregation (*Cp* and *E*_*loc*_) are key features of brain network disorganization (Jiang et al., 2020). The *Cp* reflects the degree of clustering trend of nodes. The *E*_*loc*_ represents the efficiency of information exchange in the local network, which is similar but not equivalent to its *Cp* (Latora and Marchiori, 2001; Rubinov and Sporns, 2010). In this study, relative to the young rats, the aged rats showed significantly decreased *Cp* and *E*_*loc*_ across the whole-brain metabolic network. These results were in line with previous studies reporting lower local information transfer in aged subjects. Goh (2011) demonstrated that biological aging was consistently associated with the network dedifferentiation, which was manifested as decreased intra-network connectivity, as well as increased inter-network connectivity. That is, these communities themselves in brain network are increasingly dispersed with increasing age (Bethlehem et al., 2020). This might be the main reason for the decrease of *Cp* and *E*_*loc*_ of the whole-brain metabolic network. Our findings confirmed that decreased functional segregation was also a feature of the rats’ brain metabolic network disorganization in normal aging, and further supported that the brain network tended to develop into a random network with low *Cp* during normal aging (Bullmore and Sporns, 2009).

In terms of changes in local network measures, *D* and *E*_*nod*_ are two indicators of the importance of a node (Chen et al., 2019). The *D* is the number of neighbors of the node (Bullmore and Bassett, 2011), and the *E*_*nod*_ quantifies information communication of each node within the network. Our results showed that compared with the young rats, *D* and *E*_*nod*_ of left posterior dorsal hippocampus were higher in the aged rats, indicating that the number of neighbors of left posterior dorsal hippocampus increased significantly, and its information transmission efficiency in the entire network was increased. The dorsal parts of the hippocampus receive signals from the visual, somatosensory and auditory cortices (Moser and Moser, 1998). Trompoukis et al. (2021) investigated the properties changes of synaptic transmission and neuronal excitability in the dorsal and ventral hippocampus of aged and young rats. Their research demonstrated that the dorsal hippocampus appeared able to promote transmission of low-frequency stimulus and inhibit transmission of high-frequency stimulus in young rats, while the dorsal hippocampus of old rats could not select the corresponding information transmission mode according to different frequencies. In this study, compared with the young rats, the dorsal hippocampus of the aged rats also showed significantly changes in information transmission. In addition, *E*_*nod*_ of left olfactory tubercle in the individual brain metabolic networks of the aged rats was higher, while its *D* did not change significantly. Left posterior dorsal hippocampus and left olfactory tubercle in the aged rats, as non-hubs, showed significantly increased *E*_*nod*_, which was consistent with the result of feeder connections.

Most human researches have demonstrated that the rich-club structure is a basic and common feature of large-scale brain networks that exist throughout lifespan (Colizza et al., 2006; McAuley et al., 2007). Unfortunately, abnormal rich-club organization and reduced rich-club connective strength have been observed in the human brain with normal aging (Baggio et al., 2015; Escrichs et al., 2021). Our results gave evidence to the existence of the rich-club organization in rodents’ metabolic brain networks. Depending on the hub regions, we evaluated communication efficiency in the rat metabolic networks by calculating the strength of three types of connections. The results showed that relative to the young rats, the strength of the rich-club connections decreased significantly in the aged rats, while both the strength of the feeder and local connections increased significantly. These results suggested impaired connectivity in the rich-club organization, which was consistent with the human-related studies. This may be due to the brain’s tendency to strengthen connections with non-clubs over those with rich-hubs during normal aging (Zhao et al., 2015). Given that these changes reflect increased integration and importance of non-hub regions, we speculate that our results are related to the emergence of compensatory mechanisms. In addition, these results were supported by previously reported researches suggesting that rich-club connections were more impressionable to aging (Zhao et al., 2015) and were consistent with our regional efficiency.

In addition, it is a key question to consider whether the results of animal research can be helpful for clinical intervention. At present, pharmacological therapies and rehabilitation are commonly used for the treatment of degenerative changes and diseases in the elderly. However, these approaches effect only a modest improvement (Li et al., 2020). Therefore, new non-pharmacological strategies are needed to slow age-related decline and reduce disease-related functional impairment in older adults (Sanches et al., 2021). Effective therapeutic approaches in neurodegeneration should be able to operate on the degenerative process itself or on brain plasticity (Gutchess, 2014). Non-invasive brain stimulation (NIBS) approaches have been shown to induce corrective plastic changes by targeted stimulation of different brain regions for prolonged periods (Merzenich et al., 2014). Furthermore, some studies using NIBS have shown promise improving cognitive processes related to memory and language in normal aging (Reinhart and Nguyen, 2019). Based on our findings, targeting modulation of the maladaptive brain plasticity changes, such as strengthening connectivity and network integrity in the rich-club organization and adjusting the activity intensity of brain regions involving information transmission, might be useful to promote degenerative improvement in aged adults. We hope that the findings from our preliminary study will provide foundational results to inform the target of plasticity-based neuromodulation for functional improvement in aged adults.

## Conclusion

Applying 18F-FDG PET data to explore individual brain metabolic network changes, this work provided new insights into age-related brain changes in healthy and diseased rodents. Our findings suggested abnormalities in topological properties of individual brain metabolic networks in the aged rats as well as impaired metabolic connectivity in the rich-club organization. Further research will certainly improve our understanding of brain alternations during aging.

## Data Availability Statement

The raw data supporting the conclusions of this article will be made available by the authors, without undue reservation.

## Ethics Statement

The animal study was reviewed and approved by the Animal Ethical Committee of Shanghai University of Traditional Chinese Medicine.

## Author Contributions

XX and J-JW: data analysis and manuscript draft. B-BH, X-XX, and C-LS: data collection. JM and Y-LL: data analysis. DW and Y-JD: visualization. X-YH, M-XZ, and J-GX: study design and manuscript revision. All authors read and approved the final manuscript.

## Conflict of Interest

The authors declare that the research was conducted in the absence of any commercial or financial relationships that could be construed as a potential conflict of interest.

## Publisher’s Note

All claims expressed in this article are solely those of the authors and do not necessarily represent those of their affiliated organizations, or those of the publisher, the editors and the reviewers. Any product that may be evaluated in this article, or claim that may be made by its manufacturer, is not guaranteed or endorsed by the publisher.

## Footnotes

1
http://www.fil.ion.ucl.ac.uk/spm/

2
http://www.nitrc.org/projects/gretna/
